# Perspective: How can ultrafast laser spectroscopy inform the design of new organic photoredox catalysts for chemical and materials synthesis?

**DOI:** 10.1063/1.5082620

**Published:** 2019-01-23

**Authors:** Andrew J. Orr-Ewing

**Affiliations:** School of Chemistry, University of Bristol, Cantock's Close, Bristol BS8 1TS, United Kingdom

## Abstract

Photoredox catalysis of chemical reactions, using light-activated molecules which serve as electron donors or acceptors to initiate chemical transformations under mild conditions, is finding widespread use in the synthesis of organic compounds and materials. The transition-metal-centred complexes first developed for these photoredox-catalysed applications are steadily being superseded by more sustainable and lower toxicity organic photocatalysts. While the diversity of possible structures for photoredox-active organic molecules brings benefits of design flexibility, it also presents considerable challenges for optimization of the photocatalyst molecular architecture. Transient absorption spectroscopy over timescales from the femtosecond to microsecond domains can explore the detailed mechanisms of activation and reaction of these organic photocatalysts in solution and, by linking their dynamical properties to their structures, has the potential to establish reliable design principles for future development of improved photocatalysts.

## I. INTRODUCTION

The pharmaceutical and technological benefits of sustainable synthesis of a wide range of speciality chemicals and advanced materials are driving the fast-paced development of new synthetic strategies. One powerful synthetic approach finding widespread application is to use a light-activated photoredox catalyst (PC) to drive the desired chemistry under mild conditions.^1–13^ The resulting photoredox catalytic cycles exploit low-cost near-ultraviolet (UV) and visible light sources, such as light emitting diodes (LEDs), to initiate electron-transfer reactions either from or to an excited electronic state of the molecular photocatalyst. Although the momentum for development of new photoredox-catalysed reaction schemes has largely relied on the electron donor and acceptor properties of excited states of transition-metal complexes such as [Ru(bpy)_3_]^2+^ and [Ir(ppy)_3_],^1^ there is growing interest in the development of organic molecules as photoredox catalysts.^4,14^ These organic photocatalysts (OPCs) can, in principle, circumvent the problems of toxicity, cost, and sustainability associated with many metal complexes but must contain chromophores activated by near-UV and visible light. The OPCs should have high extinction coefficients in this spectral region and must possess excited electronic states with the lifetimes and redox properties necessary to drive a range of electron-transfer reactions.

Several classes of OPCs have been developed and tested in recent years,^4,14–18^ although the arguments presented for rational OPC design differ. Much reliance remains on trial-and-error modification of OPC structures to optimize their properties for specific classes of reaction. This perspective argues that in-depth characterization of OPC excited-state properties and electron transfer reaction rates using transient spectroscopy techniques can provide a framework for more-informed design of OPCs for a range of future chemical and materials synthesis applications.

## II. PHOTOREDOX CATALYTIC CYCLES

**FIG. 1.**
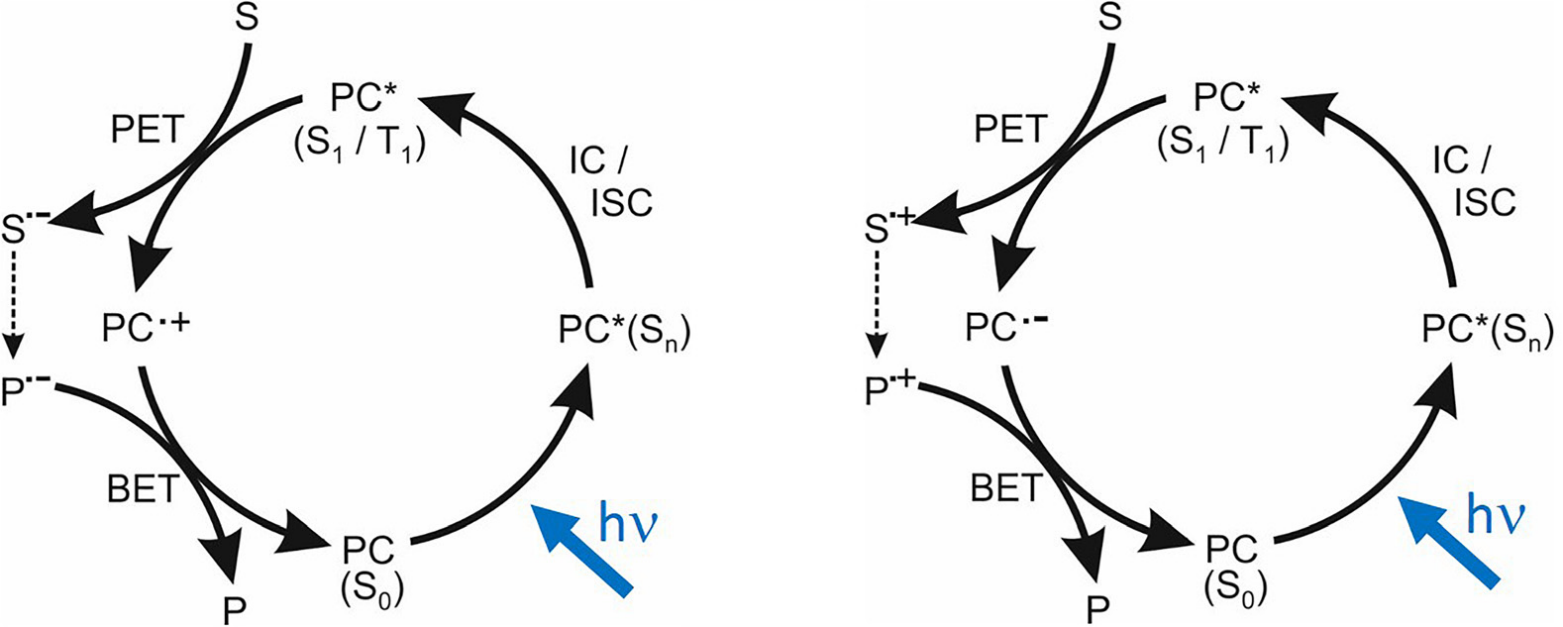
Generalized photoredox catalytic cycles. The ground electronic state photocatalyst, PC(S_0_), is photo-excited by visible or near-UV light to a higher energy singlet state (S_n_) and undergoes internal conversion (IC) or intersystem crossing (ISC) to PC* (S_1_ or T_1_) before photoinduced electron transfer (PET) with a substrate S. The left-hand cycle shows PC* as a reductant: electron donation to an acceptor S makes the oxidized PC**⋅**^+^ radical cation and a reduced substrate S·^-^ which reacts (dashed arrow) to product P⋅^-^. The right-hand cycle shows PC* as an oxidant, accepting an electron from the donor S into a vacancy in a valence molecular orbital, and reaction of the oxidized substrate S·^+^. In both cycles, back-electron transfer (BET) between reaction products P⋅^-^ or P⋅^+^ and the oxidized or reduced photocatalyst radical intermediate completes the cycle.

The principles of photoredox catalysis are illustrated by the cycles in Fig. 1, which show the two cases of the excited electronic state of the photocatalyst (denoted by PC*) acting as an outer-sphere electron donor (a reducing agent) or acceptor (an oxidizing agent) in partnership with a substrate acceptor or donor (S in Fig. 1).^1,4^ These photoinduced electron transfer (PET) processes produce either a photocatalyst radical cation (PC·^+^) and a substrate radical anion (S·^−^) or a photocatalyst radical anion (PC·^−^) and a substrate radical cation (S·^+^), respectively. Recovery of the photocatalyst in its ground electronic state (typically a singlet state, denoted by S_0_, for an OPC) involves a back-electron transfer (BET) either to PC·^+^ or from PC·^−^, with the partner in the bimolecular BET reaction being the product of the desired chemistry (P·^−^ or P·^+^ in Fig. 1). These schematic cycles are idealized representations which do not include competing processes such as PC(S_0_) recovery by back-electron transfer from S·^−^ to PC·^+^ (or PC·^−^ to S·^+^) within solvent-caged ion pairs immediately after the PET step, quenching of PC*(S_1_ or T_1_) by energy transfer to the solvent or a co-solute, or radiative decay of PC*.

**FIG. 2.**
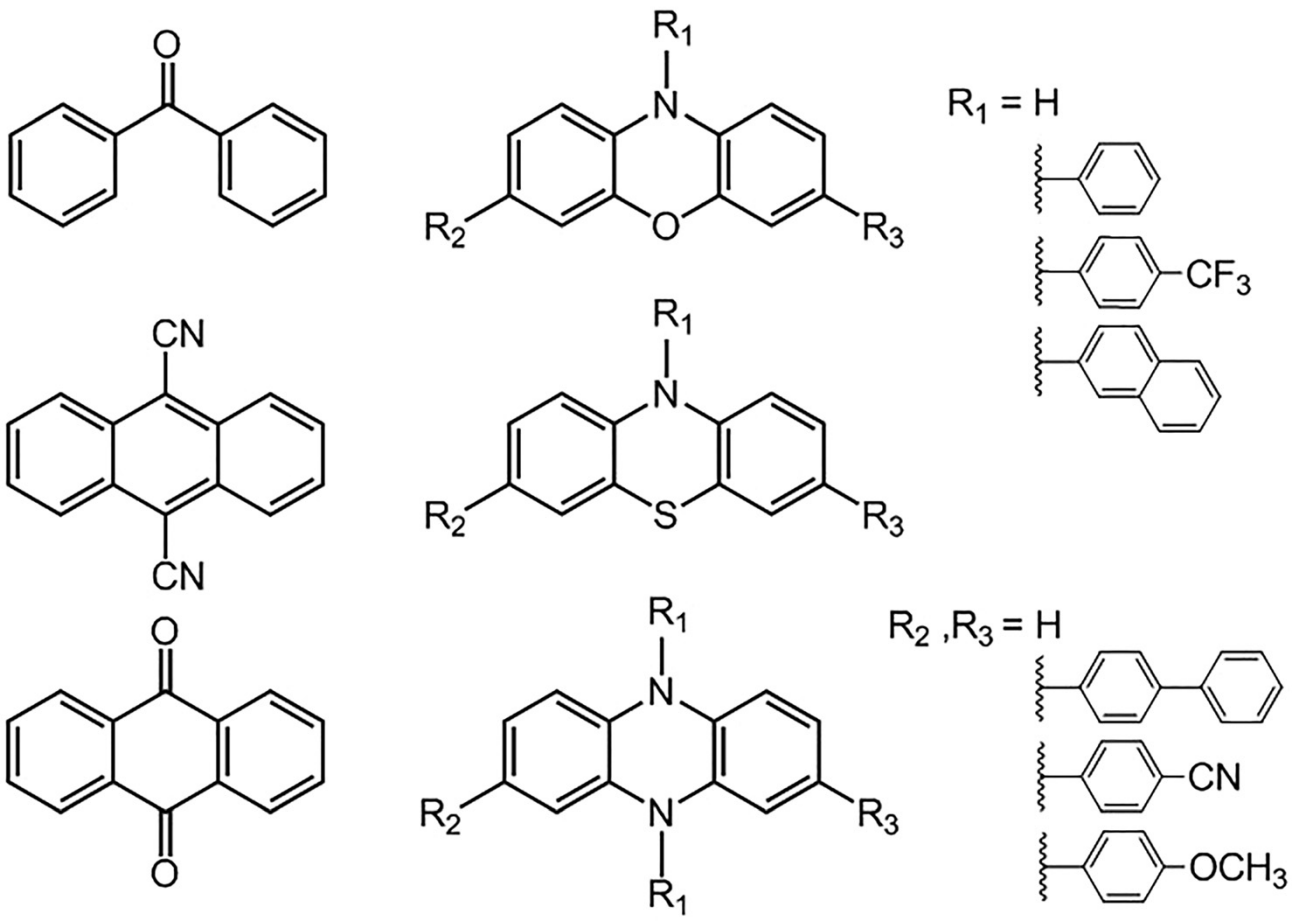
Examples of OPC structures. The first column shows three examples of commonly used OPCs, benzophenone (top), 9,10-dicyanoanthracene (middle), and anthraquinone (bottom).^4^ The second column shows OPCs based on aryl-substituted phenoxazine (top), phenothiazine (middle), and 5,10-dihydrophenazine (bottom) cores. Representative substituents at the N-atom (R_1_) and core (R_2_ and R_3_) sites are shown in the third column. Core substituents can extend the conjugation of the chromophore, and choice of both N-atom and core substituents can instill either electron withdrawing or donating character in these distinct regions of the excited state molecules.

Representative classes of OPCs are shown in Fig. 2, with the scope for further structural modification illustrated for the OPCs based on aryl-substituted phenoxazine, phenothiazine, and 5,10-dihydrophenazine cores.^14–17,19,20^ Many other examples of OPC architectures built on different core chromophore structures have also been proposed, including derivatives of xanthones, thioxanthones, xanthenes, perylenes, pyrilium ions, quinolinium ions, acridinium ions, and other organic dyes.^4^ Numerous further structures are available through modification of these various core motifs by additional functionalization, which opens up a wide space for OPC design. Initial assessment of the suitability of any given molecule as a photoredox catalyst combines absorption and emission spectroscopy methods with electrochemical techniques such as cyclic voltammetry to characterize the OPC's electronically excited states and reduction and oxidation (redox) potentials.^4^ For example, conjugated aromatic rings are common features designed into many of the OPCs, because they extend the intense π*←π absorption bands to the near-UV and visible regions which are accessible to cheap and efficient LED light sources. High triplet quantum yields are considered to be desirable targets for OPC design because a triplet spin configuration suppresses unwanted back electron transfer within the contact radical-ion pair (either [PC·^+^ S·^−^] or [PC·^−^ S·^+^]) formed by the initial electron transfer step. This back-transfer is wasteful because it recovers the ground state reactants PC(S_0_) + S; instead, solvent-cage escape and diffusive separation of the pair of radical ions are precursor steps to the intended reaction.^4^ The bimolecular electron transfer reactions in dilute solutions require diffusive encounter of reactants; hence, they further benefit from PC* triplet states with longer lifetimes than are typical for singlet excited states.

The relative merits of charge-transfer (CT) versus local-excitation (LE) character of the excited states of OPCs such as those shown in the second column of Fig. 2 have been the topic of much discussion. Arguments in favour of the benefits of excited state CT-character draw on analogies with metal-to-ligand charge-transfer (MLCT) states active in photo-induced electron transfer reactions of metal-centred complexes.^16^ Reported examples of calculated excited state electron densities for dihydrophenazine-based OPCs illustrate the concept: a strong π*←π electronic excitation on the central dihydrophenazine chromophore initially prepares a LE state with ππ* character in the centre of the molecule. Internal conversion and/or intersystem crossing can populate molecular orbitals localized on aromatic groups bound to the N-atoms, giving rise to excited states with CT character because of the depleted electron density on the dihydrophenazine core.^16^ States with CT character in the core substituent sites have also been identified in modified phenoxazine structures.^15^ Direct excitation of these CT states by absorption from the ground state is unfavourable, but their population by non-adiabatic pathways can occur on ultrafast timescales.^21^ Excited states with CT character are stabilized by polar solvents, giving characteristic Stokes shifts of their (weak) emission bands which are sensitive to the solvent polarity. Efficient population of excited CT states has been advocated as an important design objective for OPCs because they might either promote electron transfer reactions and suppress excited-state fluorescent decay^16^ or encourage intersystem crossing to triplet states.^18,22^ Drawing on both experimental data and computed molecular properties, structure-activity relationships (SARs) based on these and other arguments have been proposed as replacements for trial-and-error optimization of OPC structures, with some recent successes.^18^

## III. CHARACTERIZATION OF OPCS USING TRANSIENT ABSORPTION SPECTROSCOPY

**FIG. 3.**
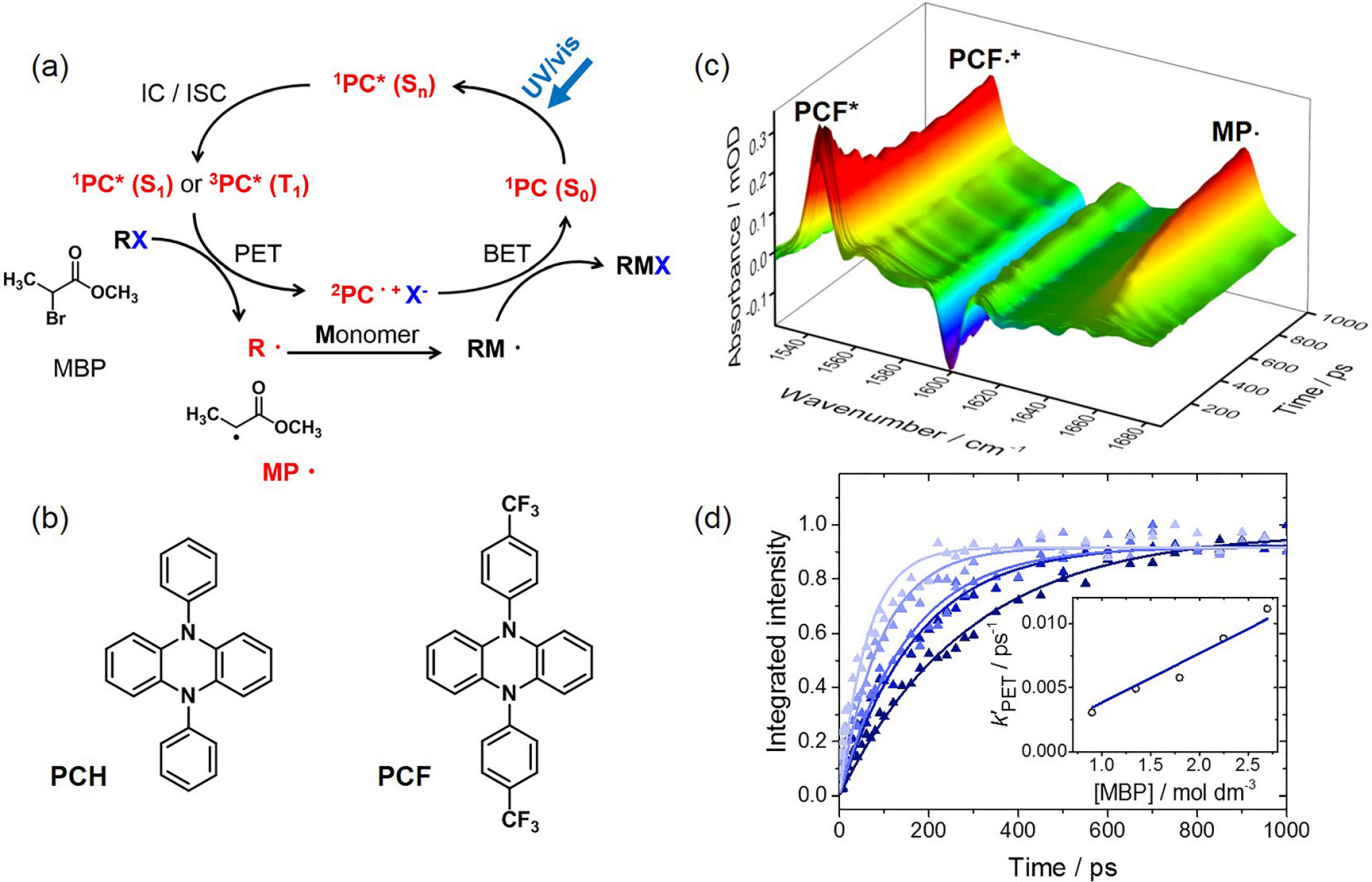
Characterization of several steps in an organocatalysed atom-transfer radical polymerization reaction using transient absorption spectroscopy.^21^ (a) The O-ATRP cycle in which MP· radicals (with the structure shown) are produced by dissociative photoinduced electron transfer (PET) from an excited state of the photocatalyst (PC) to the methyl 2-bromopropionate (MBP) initiator and react with a monomer alkene (M) to commence polymerization. All species in red have been observed by TVAS or TEAS. (b) Structures of selected aryl-substituted dihydrophenazine photocatalysts, denoted by the labels PCH and PCF. (c) Transient IR spectra of a solution of PCF and MBP in dichloromethane obtained at time delays from 1 to 1000 ps after 370-nm photoexcitation. Transient absorption features are assigned to PCF*(S_1_), PCF·^+^, and MP· radicals. (d) Time-dependence of absorption intensity on the band centred at 1660 cm^-1^, assigned to the MP· radical, for different concentrations of the MBP initiator (indicated by different coloured symbols and fitted curves). A pseudo-first order kinetic analysis is shown in the inset, from which a bimolecular rate coefficient for the PET reaction is determined.

Steady-state analytical techniques, such as the spectroscopic and electrochemical methods mentioned above, provide useful mechanistic insights and have contributed to the development of several classes of OPCs. However, much more can be learned about the mechanisms of OPC operation, and competitive processes which may reduce the efficiency of the photoredox cycles, from time-resolved dynamical studies of the OPC excited-state populations and lifetimes, and from the rates of their electron transfer reactions.^21^ These measurements can directly test hypotheses concerning the OPC-structural factors which control ISC and electron transfer rates. For example, analysis of time-evolving band intensities in transient absorption spectra can provide quantitative dynamical and kinetic data for comparison with the predictions of non-adiabatic dynamics simulations or with models based on Marcus^23–25^ (or Marcus-Savéant^26^) theory, respectively. Transient spectroscopy techniques offer a general strategy to probe structural changes in the OPCs and their co-reactants, with femtosecond to microsecond time resolution to capture the very different timescales for the stepwise processes involved in photoredox catalytic cycles. They can distinguish between, and characterize the lifetimes of, singlet and triplet states of the photoexcited OPC, as well as observe the production and loss of the OPC radical cations or anions and further radical intermediates which form by bimolecular electron transfer reactions. These measurements are best carried out in solution, using the types of solvents common in synthetic chemistry procedures, because the interactions of the photo-excited OPC molecules with the surrounding solvent can have pronounced effects on the energetic orderings of the excited states, the non-adiabatic crossing dynamics between excited states, the lifetimes of excited state populations, and the redox potentials for electron-transfer reactions. For example, the S_1_ state lifetime of 5,10-di(4-trifluoromethylphenyl)-dihydrophenazine (denoted here as PCF and with a structure shown in Fig. 3) was recently measured to be 3 ± 1 ns in dichloromethane, but only 677 ± 35 ps in *N*,*N*-dimethyl formamide (DMF). The quantum yield for intersystem crossing to populate the T_1_ state was shown to be <10% in DMF solution, with return to the S_0_ state accounting for more than 90% of the S_1_-state relaxation.^21^

Transient absorption spectroscopy of OPC solutions using pump and broad-bandwidth probe laser pulses with durations of a few tens of femtoseconds is well-suited to study both ultrafast electronically non-adiabatic excited state dynamics and bimolecular electron transfer reaction rates. Here, the focus will be on transient absorption spectroscopy in the UV/visible wavelength range, probing changes in electronic absorption spectra (transient electronic absorption spectroscopy, TEAS), and in the mid infra-red region to observe vibrational signatures of excited states and reaction intermediates (transient vibrational absorption spectroscopy, TVAS). These measurements can also distinguish photoredox catalysis by electron transfer pathways (the topic of this perspective) from photosensitization by energy transfer from the photoexcited chromophore and can explore the effects of the micro-solvation environment of the OPC on its performance. Questions about the influence of solvent-solute interactions can be addressed either by careful choice of UV excitation wavelength to excite selectively chromophores which are interacting in different ways with the surrounding solvent molecules^27^ or by analysis of the electron transfer reaction kinetics using models which incorporate diffusion.^23,28,29^ Moreover, these spectroscopic techniques can identify signatures of contact ion pairs following PET reactions^21^ and of back-electron transfer within these contact pairs prior to the desired chemical reaction. Any such back electron transfer reduces the efficiency of the photoredox cycle by regeneration of PC(S_0_) and the reagent S. Two-dimensional infra-red (2D IR) spectroscopy of the OPC solutions offers complementary information on the fluctuating interactions between solute and solvent molecules, such as the making and breaking of hydrogen bonds.^30^

The optimum design of a photoredox cycle depends, in large part, on its intended application. For example, the desirable properties of an OPC selected to enhance the yield of the products of a step in a synthetic chemical transformation may differ from those required to minimize the dispersity (and hence improve the quality) of a polymer produced by an OPC-catalysed polymerization reaction. Nocera and coworkers recently reported a painstaking mapping of the kinetics of all the productive and non-productive pathways in an Ir(III)-complex catalysed photoredox hydroamination cycle, using both spectroscopic and electrochemical methods, and showed how the efficiency of the cycle could be enhanced by informed modifications to the reaction conditions.^31^ The benefits of ultrafast transient absorption spectroscopy are illustrated by a combined TEAS and TVAS study of an organocatalysed atom-transfer radical polymerization (O-ATRP) reaction by Koyama *et al.*^21^ Contrary to expectations, this work showed that a preferred dihydrophenazine-based OPC, recommended by Miyake and coworkers on the basis of the quality of the as-grown polymer,^20^ had a low (<10%) triplet quantum yield, short S_1_ state lifetime, and relatively slow electron transfer reaction rate compared to more poorly performing aryl-substituted dihydrophenazine OPCs. These measured OPC properties apparently conflict with the usual considerations for an efficient OPC of high triplet quantum yield, long excited state lifetime, and fast electron transfer. However, the compatibility of the polymerization and the transient absorption spectroscopy studies can be understood by appreciating that maintaining a low steady-state concentration of radical species is desirable for controlled polymerization.^21,32^ The recommendations emerging from this transient spectroscopy study could therefore be tested under synthetic O-ATRP conditions by exploring the dependence of the polymer quality (e.g., its dispersity) on the concentrations of the nominally better and poorer performing OPCs. It is appropriate to emphasize that the optimum OPC properties for controlled O-ATRP are likely to differ from those required for many other synthetic chemistry targets. If rapid and efficient conversion of reactants to products is desirable, then for the reasons discussed earlier, the preferred conditions for the synthetic procedure may instead benefit from choice of OPCs which undergo fast electron transfer reactions from excited triplet states with high quantum yields.

The O-ATRP study by Koyama *et al.*^21^ serves as an instructive example of the potential for transient absorption spectroscopy to unravel the details of all the sequential steps in a multi-step photo-catalysed reaction cycle and is therefore discussed further here. It examined the properties of two *N*,*N*′-diaryl-5,10-dihydrophenazine OPCs, developed by Miyake and coworkers,^20^ and denoted here as PCF and PCH, with the structures shown in Fig. 3. This figure also shows a schematic representation of the O-ATRP cycle and examples of transient absorption spectra and kinetic measurements of electron transfer rates for experiments conducted in dichloromethane. The kinetic data were obtained by TVAS using a characteristic 1660 cm^−1^ carbonyl-stretching absorption band of the MP· radical [see Fig. 3(a)] produced by electron transfer from the OPC* to the polymerization initiator methyl 2-bromopropionate (MBP) and prompt loss of Br^−^. This band is shifted from the strong carbonyl stretch of MBP because of partial conjugation to the radical centre. With PCF as the chosen organic photocatalyst, the same kinetics also emerged from observation of the growth of an IR band at 1553 cm^−1^ and loss of a TEAS feature centred at 450 nm. The 1553 cm^−1^ band was assigned to the PCF·^+^ radical cation by steady-state IR spectroscopy, and the 450 nm band was attributed to population of the PCF(S_1_) state. Complementary characterization of the kinetics of all three chemically connected species PCF(S_1_), PCF·^+^, and MP· using a combination of TVAS and TEAS provided a comprehensive picture of the electron transfer reaction. TEAS also showed that the 450-nm absorption feature assigned to the PCF(S_1_) state developed with a time-constant of 230 ± 30 fs, most likely by internal conversion from the higher lying, optically bright S_4_ state. In the absence of the MBP initiator, the PCF(S_1_) state had a measured lifetime with respect to relaxation to the S_0_ state of only 677 ± 35 ps in DMF, which is the preferred solvent for the polymerization reactions.

There is much more that can be learned about the excited state dynamics of OPCs and about the electron transfer reactions which drive photoredox catalysis. Ongoing studies are characterizing a broad range of OPC structures to correlate triplet state quantum yields and electron transfer rate coefficients with the energies and electronic characters of the excited states involved. In accord with Marcus theory^24,25^ (or Marcus-Savéant theory for dissociative electron transfer^26^) and the Weller equation for electron transfer reactions,^33^ the electron transfer rate coefficients are expected to depend on the energies of the OPC excited states involved in PET and the PC·^+^/PC(S_0_) or PC·^−^/PC(S_0_) redox potentials. Moreover, the overall efficiency of a photoredox catalysed reaction cycle depends not only on the initial electron transfer reaction step but also on the redox potentials and rate coefficient for the back electron transfer step which returns the OPC to its ground state.^32^ The recent work by Nocera and coworkers mentioned earlier illustrates how the kinetics of these and non-reactive pathways can be pieced together by a comprehensive set of separate measurements.^31^ A collaboration between the author's research group at the University of Bristol and the Central Laser Facility at the STFC Rutherford-Appleton Laboratory is taking a different approach in its objective to provide a complete characterization of photoredox catalytic cycles. This approach seeks to quantify all the sequential reaction steps and observe the growth and decay of all reactive intermediates, in a *single* set of measurements, making use of recent technological developments in TVAS and TEAS.^34–36^ The spectroscopic measurements exploit a laser system which can make sequential transient absorption spectroscopy measurements over more than 10 orders of magnitude of time, from 10^−13^ to 10^−3^ s, following an ultrafast UV photoexcitation laser pulse.^35,36^ In this way, the evolution of excited states of the OPC (on femtosecond to few ps timescales), the initial electron transfer reactions (on ps–ns timescales), the production and reaction of radical species (on ns–*μ*s timescales), and the recovery of the OPC (S_0_) by back electron transfer (on *μ*s–ms timescales) should all be observable within a single set of transient absorption spectroscopy measurements taking only a few minutes. Using the scheme in Fig. 3 as an example, this strategy has so far been able to observe all the steps in the reactive cycle up to, and including, production of MP· radicals and their bimolecular reactions with methyl methacrylate (chosen as a representative alkene monomer). Just the final back-electron transfer reaction remains to be observed. One tell-tale signature of this final step will be the recovery of ground-state bleach features in TVAS spectra [e.g., at 1600 cm^−1^ in Fig. 3(c)], which are attributed to the initial depletion of the OPC(S_0_) state by the photoexcitation process.

## IV. CONCLUDING REMARKS

Success with the observation of the complete set of sequential steps in an OPC-catalysed photoredox cycle using transient absorption spectroscopy methods will open up many new possibilities for future investigation: the efficacies of different OPC structures can be examined quantitatively, and the outcomes combined with electronic structure calculations and kinetic models for diffusive electron transfer and radical reactions to establish robust design parameters for new OPCs; hypotheses for structure-activity relationships can be rigorously tested; and further reaction parameters can be optimized, including choice of solvent and of OPC excitation wavelength, on the basis of a molecular-level mechanistic understanding.^27^ Further advances in transient spectroscopy, for example, using emerging techniques such as ultrafast X-ray absorption spectroscopy,^37,38^ may offer additional dynamical insights, and sub-100 fs time-resolution will be necessary to observe the initial non-adiabatic dynamics in excited singlet states of OPCs and the prompt response of surrounding solvent molecules in greater detail. All these outcomes will go some considerable way to bridging the divide between the structural dynamics and synthetic organic chemistry communities.
